# Paediatric end-stage renal disease in a tertiary hospital in South West Nigeria

**DOI:** 10.1186/1471-2369-15-25

**Published:** 2014-02-03

**Authors:** Adanze O Asinobi, Adebowale D Ademola, Oluwatoyin O Ogunkunle, Susan A Mott

**Affiliations:** 1 Department of Paediatrics, College of Medicine, University of Ibadan, Ibadan, Oyo State, Nigeria; 2 Department of Paediatrics, University College Hospital Ibadan, Ibadan, Oyo State, Nigeria; 3 Centre for Chronic Disease, School of Medicine, The University of Queensland, Brisbane, Queensland, Australia

**Keywords:** End-stage renal disease, Children, Chronic kidney disease, Glomerulonephritis, Congenital anomalies of the kidneys and urinary tract, Nephrotic syndrome, Nigeria

## Abstract

**Background:**

Children and adolescents with end-stage renal disease (ESRD) in sub-Saharan Africa may have the worst outcomes globally. Barriers to management include late presentation, poor socioeconomic conditions, absence of medical insurance, limited diagnostic facilities and non-availability of chronic renal replacement therapy (RRT). Our study was to determine the incidence, aetiology, management and outcomes of paediatric ESRD in a tertiary hospital in Nigeria.

**Methods:**

A retrospective case review of paediatric ESRD at the University College Hospital Ibadan, Nigeria, over 8 years, from January 2005 to December 2012.

**Results:**

53 patients (56.6% male), median age 11 (inter quartile range 8.5-12) years were studied. Mean annual incidence of ESRD in Ibadan for children aged 14 years and below was 4 per million age related population (PMARP) while for those aged 5-14 years it was 6.0 PMARP. Glomerulonephritis was the cause in 41 (77.4%) patients amongst whom, 29 had chronic glomerulonephritis and 12 had nephrotic syndrome. Congenital anomalies of the kidneys and urinary tract (CAKUT) accounted for 11 (21.2%) cases, posterior urethral valves being the most common. Acute haemodialysis, acute peritoneal dialysis or a combination of these were performed in 33 (62.3%), 6 (11.3%) and 4 (7.5%) patients respectively. Median survival was 47 days and in-hospital mortality was 59%.

**Conclusions:**

Incidence of paediatric ESRD in Ibadan is higher than previous reports from sub-Saharan Africa. Glomerulonephritis, and then CAKUT are the most common causes. Mortality is high, primarily due to lack of resources. Preventive nephrology and chronic RRT programmes are urgently needed.

## Background

End-stage renal disease (ESRD) is an important cause of morbidity and mortality among children in sub-Saharan Africa. Managing ESRD in this region is particularly challenging due to late presentation of patients, poor socioeconomic conditions, absence of medical insurance, inadequate health care infrastructure, and poor government support. Chronic renal replacement therapy (RRT) is generally unavailable to children. Due to a combination of the afore-mentioned factors, children with ESRD in sub-Saharan Africa may have the poorest outcomes globally, even when compared with emerging economies outside Africa [1-4].

Conversely, prognosis of paediatric ESRD has improved in developed countries because most, if not all affected children can access chronic peritoneal dialysis (PD), haemodialysis (HD) or kidney transplantation [5-7]. Furthermore, there are large renal registries in North America, Europe and Australia which monitor trends in incidence, prevalence and outcomes in children with ESRD [8-11]. These registries provide valuable data for planning and delivering health services to improve the quality of life and outcomes of children with ESRD.

By contrast, paucity of such data exists in sub-Saharan Africa [1,2,12,13], without which, advocacy, development of intervention strategies, and evaluation of effectiveness of interventions are restricted. This study aims to make a contribution by determining current incidence, aetiology, management and outcomes of paediatric ESRD in a tertiary hospital in Nigeria.

## Methods

The University College Hospital Ibadan is a large tertiary care referral centre located in Ibadan, the capital city of Oyo State, in South West, Nigeria. Patients come from Ibadan, other parts of Oyo State and beyond. Approximately 68% of the population of Nigeria live below the United Nations Children’s Fund (UNICEF) poverty line of 1.25 dollars per day [14]. Patients are required to pay out-of-pocket for medical care, including RRT. The National Health Insurance is limited to a minority of employees of the Federal Government as of now and does not include the cost of dialysis or kidney transplantation.

According to the 2006 national census, Oyo State has a population of 5,580,894 and a growth rate of 3.35%. The population of children aged ≤ 14 years in Oyo state is 2,099,694, while that of children aged 5-14 years is 1,385,660. While Ibadan, the capital city, has a population of 2,560,573 with populations of children aged ≤ 14 years and those aged between 5-14 years in Ibadan 949,639 and 608,012 respectively [15-17].

The hospital’s paediatric nephrology unit is the only one in Oyo state. It offers acute peritoneal dialysis with rigid or adapted catheters and acute haemodialysis [18]. Chronic dialysis is not available due to barriers related to cost. Kidney transplantation have recently become available in the adult nephrology unit (four have been performed) and there are ongoing efforts to extend these services to children.

As a retrospective study, case records of all the patients who were managed for ESRD in the Paediatric Nephrology Unit of the hospital from January 2005 to December 2012 (96 months) were reviewed. Demographic, clinical, anthropometric, laboratory, aetiology of ESRD, and RRT modality data were extracted and entered into a questionnaire and subsequently into the Statistical Package for Social Sciences (SPSS) version 17 (Reproduced 2008, SPSS Statistics for Windows, Version 17. Chicago: SPSS Inc.) spread sheet.

ESRD was defined as need for dialysis, commencement of dialysis or death from renal failure in patients with chronic kidney disease (CKD) [19]. Chronic kidney disease was defined as kidney damage or glomerular filtration rate of <60 ml/min/1.73 m^2^ for ≥ 3 months, and kidney damage was defined as pathologic abnormalities or markers of damage on urine, blood or imaging tests [19]. For patients who presented to us in ESRD and were followed up for less than 3 months, the diagnosis of background CKD was made from the patient’s clinical history or if the patient was stunted (height < 3^rd^ percentile) [20]. Chronic Kidney disease was also diagnosed if the patient had congenital anomalies of the kidneys and urinary tract (CAKUT) or ultrasound finding of shrunken kidneys [19,21]. Nephrotic syndrome was defined as massive proteinuria (dipstick urinalysis ≥ 3+ proteinuria or 24 hour urinary protein of 40 mg/m^2^/hour) with serum albumin of <2.5 g/dl, oedema and hyperlipidaemia [22]. Chronic glomerulonephritis (CGN) was characterized by hypertension, renal failure, proteinuria and haematuria. The term glomerulonephritis referred to nephrotic syndrome or CGN [22] CAKUT were diagnosed on the basis of ultrasound, micturating cysthourethrogram, or autopsy findings.

A primary outcome measure of in-hospital mortality was determined as a recording of mortality was only available for patients who died while admitted. The secondary outcome measure was the duration of survival in ESRD. Mean or median and inter quartile range (IQR) was calculated for continuous variables. Proportions were determined for categorical variables. The numerator for annual incidence of ESRD was children who reached ESRD in the respective year, who were domiciled in Ibadan and were managed in our hospital. The denominator for the incidence of ESRD incidence was the age related population of children in Ibadan for the year based on the 2006 national census [15-17]. Incidence of ESRD was not calculated for children aged 15 years and above because patients in this age group variably present to and are managed in the adult nephrology unit. Data of patients managed in the adult unit were not included in this study.

Kaplan Meier survival analysis was used to determine the median survival time from the day of presentation in ESRD to in-hospital mortality. Patients who were not hospitalized or who were discharged home were censored. Cox proportional hazard regression analysis model was used to assess the effect of gender, age, presentation in ESRD, aetiology of ESRD and acute dialysis, on duration of survival in ESRD. For this analysis, age was grouped as 9 years and below versus 10 years and above; and aetiology of ESRD was grouped as CAKUT and acquired (glomerulonephritis and malignancy) causes. Patients were also grouped into those who received any form of dialysis and those who did not. Statistical analysis was done with the SPSS version 17 software.

Ethical approval for the study was obtained from the University of Ibadan/University College Hospital (UI/UCH) Ethics Committee and the study was performed in line with the Declaration of Helsinki.

## Results

### Demographics

A total of 53 patients in ESRD, aged between 18 days and 17 years, were seen over a period of 96 months. There were 30 males (56.6%). The median age at presentation in ESRD was 11 (IQR 8.5-12) years. Four patients (7.5%) were < 5 years, two of whom were less than 1 year old. Fourteen patients (26.4%) were aged 5-9 years, while 28 (52.8%) were aged 10-14 years (See Table 1).

**Table 1:** Review characteristics for children and adolescents with ESRD: University College Hospital Ibadan, Nigeria, 2005-2012

**Age group, (years)**	**Gender**	**Diagnosis**			**ESRD at initial presentation (N)**	**Acute dialysis**				**In-hospital mortality (%)**	**N (%)**
		**GN**	**CAKUT**	**Malig**		**HD alone**	**PD alone**	**HD and PD**	**None**		
0-4	F	-	1	-	1	-	1	-	-	1	1
	M	-	3	-	3	-	3	-	-	3	3
*Subtotal*		*-*	*4*	*-*	*4*	*-*	*4*	*-*	*-*	*4*	*4 (7.5)*
5-9	F	6	1	-	4	3	2	-	2	3	7
	M	5	2	-	5	7	-	-	-	1	7
*Subtotal*		*11*	*3*	*-*	*9*	*10*	*2*	*-*	*2*	*4*	*14 (26.4)*
10-14	F	13	-	-	8	9	-	2	2	9	13
	M	12	3	-	9	9	-	1	5	10	15
*Subtotal*		*25*	*3*	*-*	*17*	*18*	*-*	*3*	*7*	*19*	*28 (52.8)*
15-19	F	-	1	1	1	2	-	-	-	1	2
	M	5	0	-	3	3	-	1	1	1	5
*Subtotal*		*5*	*1*	*1*	*4*	*5*	*-*	*1*	*1*	*2*	*7 (13.2)*
**Total N (%)**		**41 (77.4)**	**11 (20.8)**	**1 (1.8)**	**34 (64.2)**	**33 (62.3)**	**6 (11.3)**	**4 (7.5)**	**10 (18.9)**	**29 (54.7)**	**53 (100)**

CAKUT: Congenital Anomalies of the Kidneys and Urinary Tract; ESRD: End-stage renal disease; GN: Glomerulonephritis; F: Female; M: Male; HD: Haemodialysis; Malig: Malignancy; PD: Peritoneal dialysis.

**Figure 1:**
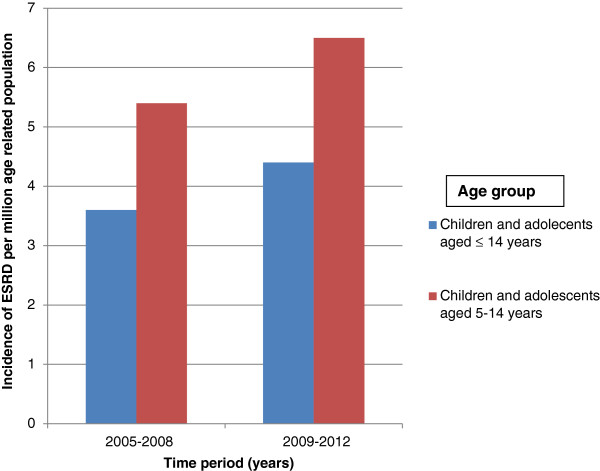
Incidence, per million age related population, of end-stage renal disease (ESRD) for children and adolescents, by age group and time period: University College Hospital Ibadan, Nigeria.

Thirty six patients (67.9%) lived in Ibadan, one (1.9%) was from a town outside Ibadan but within Oyo state, while the other 16 (30.2%) were from other states in the country. Among the patients from Ibadan 32 (91.4%) were aged 14 years and below, while 31 (86.1%) were aged 5-14 years. The mean annual incidence of ESRD among children aged 14 years and below who were domiciled in Ibadan was 4.0 per million age related population (PMARP) while the mean incidence for children aged 5-14 years in Ibadan was 6.0 PMARP. The mean incidence of ESRD for the years 2005 to 2008 and 2009-2012 among children aged ≤ 14 years were 3.6 and 4.4 PMARP respectively, while ESRD incidence for children aged 5 - 14 years were 5.4 and 6.5 PMARP (See Figure 1).

Nineteen patients (35.8%) presented before the onset of ESRD and subsequently progressed to ESRD; while 34 (64.2%) were already in established ESRD at the time of initial presentation.

### Aetiology of ESRD

Regarding children who presented before the onset of ESRD, 11 (57.9%) presented with nephrotic syndrome, while 5 (26.3%) presented with CGN. One of the patients with nephrotic syndrome also had a solitary kidney. Cyanotic congenital heart disease, sickle cell anaemia and HIV infection were the initial presentation in three patients.

Overall, ESRD was due to glomerulonephritis in 41 children (77.4%), with CGN and nephrotic syndrome occurring in 29 (54.7%) and 12 (22.6%) of children respectively. Seropositivity for HBsAg and HIV infection was associated with glomerulonephritis in four and three children respectively (See Table 2).

**Table 2:** Pattern of aetiology of end-stage renal disease and associated features among affected children and adolescents

**Diagnosis**		**N (%)**	
Glomerulonephritis	Chronic glomerulonephritis (Non-nephrotic)	29 (54.7)	
	Unknown aetiology, n = 23		
	HIV seropositivity, n = 3		
	HBsAg seropositivity, n = 2		
	Sickle Cell Nephropathy, n = 1		
	Nephrotic syndrome	12 (22.6)	
	Unknown aetiology, n = 7		
	FSGS, n = 2		
	HBsAg seropositivity, n = 2		
	CCHDX (Tricuspid atresia), n = 1		
	*Subtotal*		*41 (77.4)*
Congenital anomalies of the kidney and urinary tract	PUV	6 (11.3)	
	Right sided solitary kidney^a^	2 (3.8)	
	Others	3 (5.7)	
	*Subtotal*		*11 (20.8)*
Malignancy	Bilateral non-Hodgkin’s lymphoma of the kidneys	1 (1.9)	
	*Subtotal*		*1 (1.9)*
*Total*		53 (100.0)	

^a^One of the patients with right sided solitary kidneys presented with nephrotic syndrome.

CCHDX: Cyanotic congenital heart disease; FSGS: Focal segmental glomerulosclerosis; HBsAg: Hepatitis B surface antigen; HIV: Human immunodeficiency virus; HIVAN: Human immunodeficiency virus-1-associated nephropathy; PUV: Posterior urethral valves.

Renal biopsy was performed in seven children and showed focal segmental glomerulosclerosis (FSGS) in four patients. Diffuse global sclerosis, membranous nephropathy and non-Hodgkin lymphoma of the kidneys were observed in one patient each.

CAKUT was associated with ESRD in 11 patients (20.8%). Five of these had posterior urethral valves (PUV), while 2 had solitary kidney (See Table 2). Ten of the 11 patients with CAKUT were already in established ESRD at initial presentation to our facility. Apart from sickle cell nephropathy, hereditary nephropathy was not recorded in any child.

### Management

#### Immunosuppressive treatment

Among the 11 children with nephrotic syndrome who presented before the onset of ESRD, 6 had received treatment with corticosteroids and were all steroid resistant. Three children defaulted from follow up before commencement of steroid therapy and later represented in ESRD. The patient with nephrotic syndrome, cyanotic congenital heart disease and polycythaemia was not offered corticosteroids. The one with nephrotic syndrome and solitary kidney had associated hypertension requiring the use of combination anti hypertensive therapy and also did not receive corticosteroid or other immunosuppressive therapy.

#### Renal replacement therapy

Fifty one children were admitted into the hospital for the management of ESRD, while 2 were managed on outpatient basis only. Forty three children (81.8%) altogether underwent acute dialysis. Thirty three patients (62.3%) had intermittent HD alone, 6 (11.3%) had acute PD alone, while 4 children (7.5%) underwent both intermittent HD and acute PD (See Table 1). Two patients received one session each of HD on out-patient basis following discharge from the hospital, otherwise dialysis took place while patients were on admission. One of the patients with PUV proceeded to have valve ablation and kidney transplantation in India. Chronic renal replacement therapy could not be carried out in the other patients because of financial constraints.

#### Outcome

**Figure 2:**
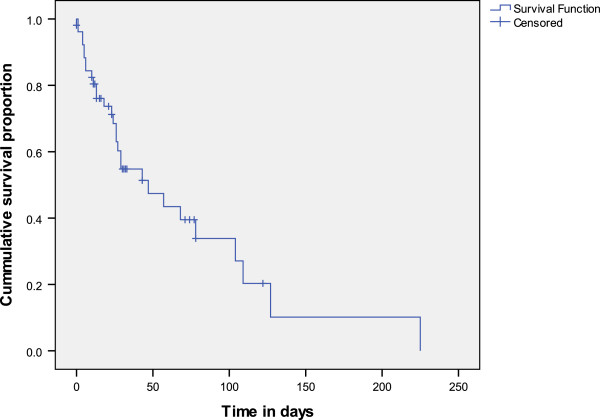
Kaplan Meir survival curve of time from presentation in end-stage renal disease to in-hospital mortality, among 53 children and adolescents: University College Hospital Ibadan, Nigeria, 2005-2012.

**Figure 3:**
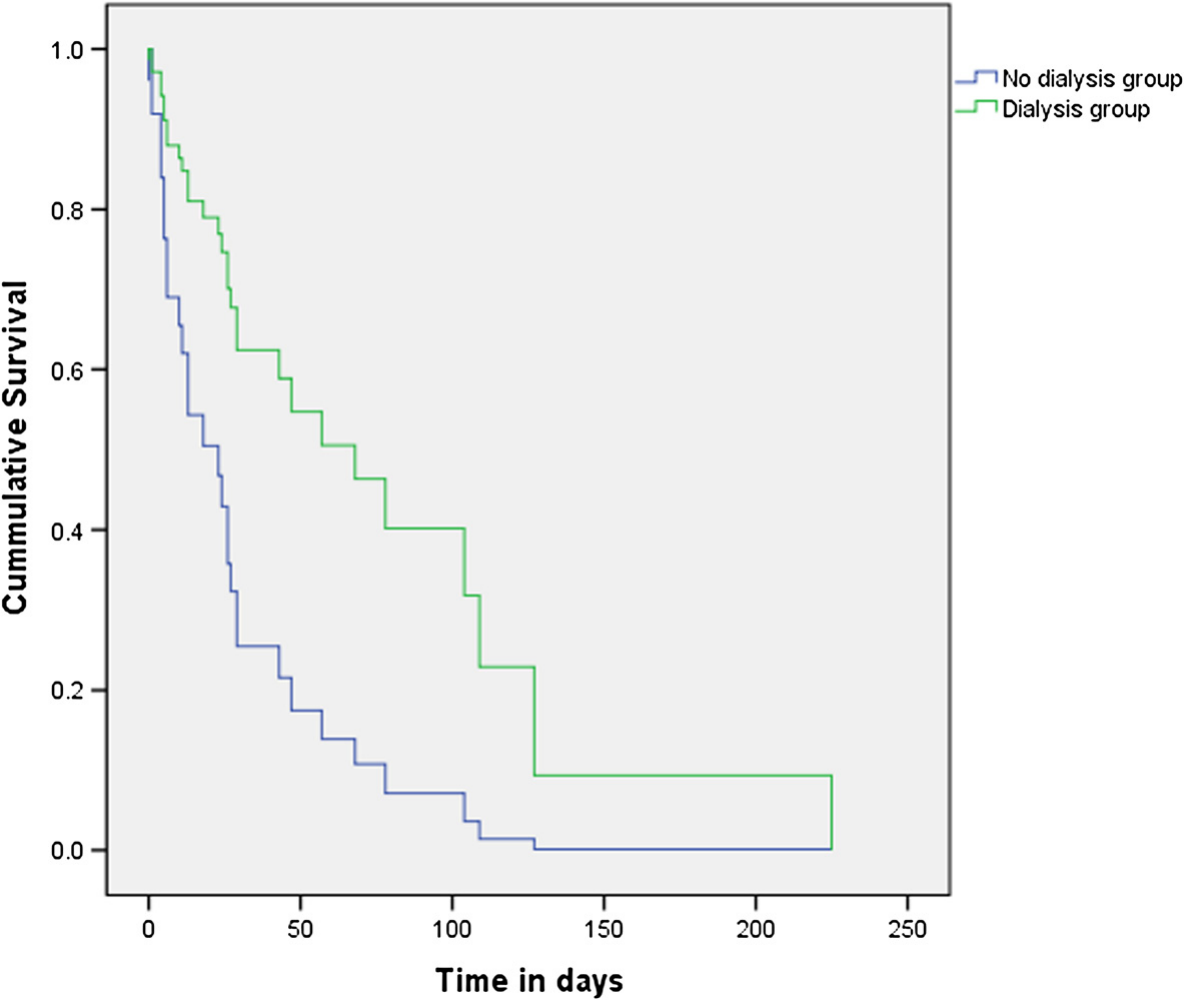
Plot of survival of 53 children and adolescents from presentation in ESRD to in-hospital mortality, by acute dialysis management: University College Hospital Ibadan, Nigeria, 2005-2012.

For the 19 patients who had presented before developing ESRD, the time from initial presentation to presentation in ESRD was a median of 28 (IQR 5 – 38) months. Fifty one patients were hospitalised for ESRD management, out of which 29 (59%) died in the hospital. The time from presentation in ESRD to in-hospital mortality ranged from 8 hours to 7.5 months, and median survival was 47 (SE 15) days (See Figure 2). The Cox Proportional Hazard regression analysis (Figure 3) showed that patients who did not receive dialysis survived for significantly less number of days than patients who did (p = 0.042, Hazard ratio: 2.9, 95% Confidence interval 1.039-8.083). Age, gender, aetiology, and presentation in ESRD did not have significant effect on survival when patients were in ESRD.

## Discussion

Children in ESRD are the most severely ill in the spectrum of childhood chronic kidney disease (CKD), and carry the highest risk for mortality. There are few studies on childhood ESRD from sub-Saharan Africa [1,2,12,13]. To the best of our knowledge none of the studies were reported exclusively on children in ESRD and therefore, our study adds important new information to the epidemiology of ESRD among Nigerian children. Our study showed that the incidence of paediatric ESRD was higher than previously reported from our sub region [1,2,13] but lower than those reported in developed countries [8,9,11,23]. Glomerulonephritis was the commonest cause, followed by CAKUT. Management of the patients was hampered by lack of funds and facilities for chronic RRT resulting in high mortality.

This study noted a variation in the distribution of ESRD within Oyo state with virtually all patients domiciled in the state, coming from within Ibadan, the capital city, where our hospital is located. This variation is most likely due to differences in referral patterns of ESRD within the state. Awareness, recognition and referral of children with ESRD may be higher from communities that are closer to the University College Hospital which is the sole centre offering paediatric dialysis services in Oyo State. Variation in reporting of ESRD by accessibility to health services has been previously documented with higher reporting from metropolitan areas noted in Jamaica and Chile [4,24]. Concentration of main health resources and technology for the diagnosis and treatment of renal diseases in metropolitan areas and under reporting of kidney disease from other areas are assumed to be reasons for differential referral patterns [4,24].

The few studies on childhood ESRD from sub-Saharan Africa, mainly from Nigeria [1,2] and South Africa [13], also included patients with other stages of CKD and found a CKD incidence of 1-3 per million children. The present study found an ESRD incidence of 4 per million children in Ibadan which was higher than findings from other parts of Nigeria [1,2] or South Africa [13]. The higher incidence of ESRD in our study compared to the other Nigerian studies may be due to differences in referral patterns, while the higher incidence compared to findings in South Africa may be related to variations in genetic or environmental factors.

Additionally we found an increasing incidence of paediatric ESRD over the 4 year bands 2005-2008, and 2009-2012. This is similar to a report from the United Kingdom which also noted a rising incidence in paediatric RRT for ESRD over 3 consecutive 5 year bands from 1996-2010 [25]. The rising incidence of RRT in the United Kingdom was attributed to increased provision of dialysis to younger children due to improvements in techniques for nutritional and dialysis support and increasing referral of children who are younger than 16 years of age to paediatric units [25]. The rising incidence of ESRD in the present study may be due to increasing recognition and referral of children in ESRD.

The incidence of childhood ESRD observed in this study is lower than that reported from many developed countries. In the United States and Canada an ESRD incidence of 9-16 per million is recorded for children aged 0-19 years [11]. The North American registries included patients in the 15-19 year age group an age group reportedly having the highest incidence of paediatric ESRD [9,11,23]. The incidence in Ibadan was also less than the mean of 5.3 per million for children less than 14 years old reported in European countries [8], or 10.9 per million children as noted in Turkey [26]. It was however much less than the 17 per million children noted in Kuwait in the Middle East, where hereditary renal diseases are common due to high prevalence of consanguinity [27]. Poor health seeking behaviour, under-reporting of cases and referral patterns in poor resource settings may also contribute to the low incidence in Ibadan compared to developed nations. Additionally, the lack of resources for appropriate consultations, medications, chronic dialysis and kidney transplantation for children accelerates mortality in patients with both acute and chronic kidney diseases leading to a lower incidence and prevalence of ESRD in Ibadan and indeed in many parts of sub-Saharan Africa.

The male predominance noted in this study is similar to the observation in many other reports [11,23,25-29] including studies done in Africa [1,2,12,13]. The male predominance, as in the other studies, is due to the higher proportion of males among patients with CAKUT. Furthermore, there are relatively few patients in the 0-5 year age group in our study compared to other age groups in our study. ESRD in younger children is mostly due to CAKUT, while the contribution of glomerulonephritis to ESRD increases with age [1,9,25,30,31]. The low number of patients who are less than 5 years of age in our study may reflect the finding of low numbers of patients with CAKUT versus those with glomerulonephritis.

Glomerulonephritis was the leading cause of ESRD in our study, accounting for approximately 80% of cases. This was followed by CAKUT in approximately 20% of our patients. This is in contrast to reports from Europe [8], Australia [9], Japan [23], Kuwait [27], Turkey [26] and among whites in the United States [11] where CAKUT are the largest causes of ESRD and are responsible for 34% - 52% of cases of ESRD among children. Glomerulonephritis accounts for 14 - 30% childhood ESRD in these settings [8,9,11,23,27]. Among African American children primary and secondary glomerulonephritis were the leading cause of ESRD [11]. Reports from India, South East Asia and South America indicate that glomerulonephritis is the predominant cause of childhood CKD in developing countries [3,32-34]. Similarly in a South African study [13] with 75% black participants and in other studies from sub-Saharan Africa [1,2,12], glomerulonephritis was the largest cause of CKD occurring in 25.4 - 56.4% of children, while CAKUT occurred in 17.5-37.3% of children [1,2,10,11]. The predominance of glomerulonephritis followed by CAKUT among our patients is consistent with the observation in African Americans and in developing countries including those in sub-Saharan Africa [1,2,11-13].

The large proportion of patients with glomerulonephritis compared to CAKUT in this study may be due to both genetic and environmental factors. The renal biopsies, although few were performed, predominantly showed FSGS. Among African American children, FSGS is three times more common than in Caucasian children, suggesting a genetic basis [10,28]. In Ibadan, where our hospital is located, a strong association was previously found between APOL 1 variants and their two allele gene haplotypes in a study of 166 adult Nigerian patients with non diabetic kidney disease [35]. Furthermore, vesico-ureteral reflux, a congenital anomaly which is commonly associated with renal hypoplasia and dysplasia and reflux nephropathy among children in Europe, Asia, and the Middle East and also among white Americans is less common among African Americans and in sub-Saharan Africa [1,2,12,36-39].

The high prevalence of glomerulonephritis among children in developing countries and in sub-Saharan Africa has also been attributed to the high prevalence of bacterial, protozoal or viral infections [40,41]. In our study 20% of the patients with glomerulonephritis were either seropositive for HIV or Hepatitis B surface antigen. With increasing survival of HIV infected children and reduction in mortality from infectious diseases, HIV appears to be emerging as a cause of paediatric ESRD [42-44]. It is not clear, however, if Hepatitis B virus infection was the aetiology of ESRD in our patients or if it was incidental [45].

The high proportion of patients with glomerulonephritis among our patients compared with CAKUT might also be due to patient selection. Our study evaluated only children in ESRD. It is recognised that glomerulonephritis progresses to ESRD faster than CAKUT [36,46]. Additionally severe cases of CAKUT may have been missed as they might have died in infancy or early childhood without diagnosis.

The majority (60%) of our patients already had ESRD at the time of their initial presentation. This pattern of late presentation has been noted in developing countries like India [31,32], Jamaica [4] and Syria [47], where ESRD was present in 20-55.5% of children with CKD at presentation. Late presentation has also been noted among ethnic minorities in the UK [48]. In contrast, among children in the North American Renal Transplant and Cooperative Study (NAPRTCS), with many of the contributing centres in developed countries, only 4.3% were in ESRD at entry into the study [10]. The early features of CKD may not be overt and may have been missed by the caregivers leading to late presentation in some of our patients. Furthermore there may be low priority for screening and referral of children for kidney disease in many primary and secondary health facilities in Nigeria because chronic renal replacement is expensive and not readily available. Early diagnosis of kidney disease may, however, allow institution of therapy that will potentially prevent or slow the progression to ESRD [22]. Efforts to increase awareness of childhood kidney disease among the public and health workers, to promote early diagnosis, referral and treatment of kidney disease among children are needed.

Access to chronic RRT has improved the outcome of ESRD in developed countries. The case fatality ratio decreased from 11.0 per 100 patient years in the 1960s to 1.3 per 100 patient years in 2002-2004 [7,49]. Long term survival rate for children on chronic RRT in developed countries is 79% at 10 years and 66% at 20 years [6,28]. Transplant patients consistently have a 4 fold survival advantage compared with dialysis patients [6,11]. Life expectancy among young adults who started RRT in childhood is 38 years for patients remaining on dialysis compared to 68 years for those with a functioning graft [50]. In developed countries, deaths in children from ESRD now occur mainly from infections and cardiovascular disease rather than renal failure in contrast to the pattern in developing countries [28]. Although we offered acute dialysis to more patients in ESRD than in previous Nigerian reports on paediatric ESRD, in the absence of chronic RRT outcome remained very poor. Patients who were discharged home would have died at home shortly after discharge in the absence of chronic RRT. Similar high mortality was noted in previous reports from Nigeria [1,2]. Some centres in the country, including ours, have started performing kidney transplantation, the financial implication (including cost of immunosuppressive medications) is, however, prohibitive for most Nigerians. The absence of chronic renal replacement therapy was the cause of the high mortality in our patients.

Our study has a number of limitations, one of which is that it reflects a single centre hospital based incidence and the data is likely an underestimate of the true incidence of ESRD since many patients may not present or continue follow up at a tertiary care centre. A prospective multicentre study may give a better estimate, although prospective community based study will be ideal. Another limitation of our study is that the aetiology of chronic glomerulonephritis was not determined in many of our patients and that few renal biopsies were performed. In sub-Saharan Africa late presentation and limited diagnostic facilities, including facilities for immunofluorescence and electron microscopy of renal biopsies, make it difficult to determine the aetiology of glomerulonephritis in many patients. Multicentre and prospective studies are needed to accurately determine the aetiology of chronic glomerulonephritis in sub-Saharan Africa. The study however indicates that the incidence of paediatric ESRD in the sub-region may be higher than previously reported and underscores the high mortality associated with this condition in sub-Saharan Africa.

In view of the high mortality associated with ESRD in settings with very limited access to chronic RRT, urgent steps need to be taken in the areas of preventive nephrology, development of a public-funded chronic RRT program, and setting up of a national registry for paediatric ESRD. Programs that increase awareness of kidney disease among the public and health workers may lead to earlier presentation, detection and management of kidney disease. Optimal management of patients with glomerulonephritis is needed with adequate laboratory back up and access to medications. Early diagnosis and appropriate management of children with CAKUT may reduce mortality; it is however unclear if management will improve long term renal outcomes especially with posterior urethral valves [51,52]. A public-funded chronic RRT program is therefore needed to improve access to dialysis and kidney transplantation and potentially lead to improved prognosis. Nigerian nephrologists and other stake holders should continue to provide advocacy for the National Health Insurance to be expanded to include a larger proportion of the population and the cost of chronic RRT. The setting up of a national registry of paediatric ESRD will provide important data for advocacy, to monitor outcome of interventions and national trends in paediatric ESRD. In addition to governmental support, that from non-governmental bodies and individuals is required to achieve sustained improvement of paediatric nephrology services and research in Nigeria and sub-Saharan Africa.

## Conclusion

In conclusion, the incidence of ESRD in our study is higher than in previous Nigerian studies. Glomerulonephritis is the commonest cause followed by CAKUT. Prognosis remains poor in the absence of chronic RRT. Preventive nephrology and public-funded chronic RRT programmes are urgently needed.

## Competing interests

The authors declare that they have no competing interests.

## Authors’ contributions

AOA: Conceptualized the work, data interpretation, and revised the article critically for intellectual content. ADA: Performed data analysis and interpretation. OOO: Performed data interpretation and revised the article critically for the intellectual content. SAM: Performed data interpretation and revised the article critically for the intellectual content. All the authors approved the final version.

## Authors’ information

AOA: Lecturer and Honorary Consultant Paediatric Nephrologist. ADA: Lecturer and Honorary Consultant Paediatric Nephrologist. OOO: Lecturer and Honorary Consultant Paediatrician. SAM: Research Officer.

## Pre-publication history

The pre-publication history for this paper can be accessed here:

http://www.biomedcentral.com/1471-2369/15/25/prepub
